# CRAFITY score benefits hepatocellular carcinoma patients treated with transarterial chemoembolization and lenvatinib

**DOI:** 10.1002/cam4.7410

**Published:** 2024-06-24

**Authors:** Lin Zhang, Hongcai Yang, Shangkun Ning, Zhijuan Wu, Dianzhe Wang, Hexin Liang, Chunni Wang, Xu Chang

**Affiliations:** ^1^ Department of Interventional Therapy I, Shandong Cancer Hospital and Institute Shandong First Medical University and Shandong Academy of Medical Sciences Jinan Shandong China; ^2^ Department of Interventional Therapy, National Cancer Center/National Clinical Research Center for Cancer/Cancer Hospital Chinese Academy of Medical Sciences and Peking Union Medical College Beijing China; ^3^ Department of gerontology Central Hospital Affiliated to Shandong First Medical University Jinan Shandong China; ^4^ Department of Radiology, Shandong Cancer Hospital and Institute Shandong First Medical University and Shandong Academy of Medical Sciences Jinan Shandong China; ^5^ Department of Radiation Oncology, Shandong Cancer Hospital and Institute Shandong First Medical University and Shandong Academy of Medical Sciences Jinan Shandong China; ^6^ Department of Interventional Therapy II, Shandong Cancer Hospital and Institute Shandong First Medical University and Shandong Academy of Medical Sciences Jinan Shandong China

**Keywords:** alpha‐fetoprotein, CRAFITY score, C‐reactive protein, hepatocellular carcinoma, Lenvatinib, TACE

## Abstract

**Background:**

The CRAFITY score serves as a simple and effective predictive model for individuals diagnosed with hepatocellular carcinoma (HCC) and subjected to treatment with atezolizumab and bevacizumab (Atez/Bev). However, no large sample size studies have reported the application of the CRAFITY score among HCC patients undergoing transarterial chemoembolization (TACE) in conjunction with lenvatinib. This research aims to assess the prognostic role of the CRAFITY score in the context of individuals with HCC receiving TACE in combination with lenvatinib.

**Methods:**

This retrospective analysis encompassed 314 individuals diagnosed with HCC who underwent the combination of TACE and lenvatinib at two medical facilities in China from August 2019 to August 2022 (comprising a training cohort of *n* = 172 and a validation cohort of *n* = 142). We investigated the prognostic values of overall survival (OS), progression‐free survival (PFS), disease control rate, and objective response rate in the training cohort based on the CRAFITY scores. Furthermore, the predictive capacity of the model was corroborated through validation using an external cohort.

**Results:**

We included 174 and 142 patients treated with TACE plus lenvatinib in the training and validation cohorts, correspondingly. PFS and OS differed across all three groups in all training and validation cohorts, based on the CRAFITY score (*p* < 0.001). In both cohorts, the CRAFITY score effectively predicted tumor response (*p* < 0.001). Moreover, among the 121 patients who received TACE, lenvatinib, and immunotherapy, the CRAFITY score showed promising predictive efficacy in PFS and OS.

**Conclusions:**

The CRAFITY score, utilizing C‐reactive protein and alpha‐fetoprotein values, emerges as a dependable and pragmatic instrument for forecasting the effectiveness of TACE plus lenvatinib in individuals with unresectable HCC. This scoring system holds the potential to assist oncologists in making informed clinical decisions.

## Graphical abstract

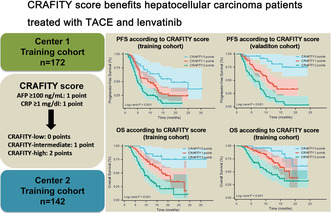

## 1 INTRODUCTION

Hepatocellular carcinoma (HCC) stands as one of the prevalent malignancies globally, constituting a primary contributor to cancer‐related fatalities.^1^, ^2^ In China, a substantial number of individuals with HCC receive diagnoses at advanced stages (in local cases) or with distant metastases. This can be attributed to the insidious onset, elevated invasiveness, rapid progression, and challenges associated with early detection of HCC, ultimately limiting the potential for surgical resection opportunities.^3^, ^4^ The evolution of targeted therapy and immunotherapy has prompted a shift in the approach to treating advanced HCC, transitioning from local treatment to comprehensive management through combined systemic treatment. Notably, transarterial chemoembolization (TACE) combined with lenvatinib has become an important treatment option.^5^, ^6^ Nevertheless, the reported outcomes of HCC treatment utilizing TACE in conjunction with lenvatinib vary among different medical centers.^5^, ^6^, ^7^, ^8^

Several prognostic models, as reported in the literature, have demonstrated encouraging predictive capabilities in assessing which patients benefit more from TACE or lenvatinib for HCC.^9^, ^10^, ^11^, ^12^, ^13^ Unfortunately, there is currently a lack of established models specifically designed to predict the prognosis of patients undergoing treatment with TACE combined with lenvatinib. Consequently, developing a simple, effective, and accurate evaluation method for combined treatments is imperative.

Scheiner et al.^14^ have introduced a novel prognostic scoring system termed “CRAFITY,” formulated on the basis of serum levels of alpha‐fetoprotein (AFP) and C‐reactive protein (CRP). This scoring system facilitates the straightforward categorization of patients with HCC into three distinct prognostic strata. Lower CRAFITY scores correlate with more favorable tumor response and improved overall survival (OS).

While existing research has highlighted the efficacy of CRAFITY scores in predicting OS among HCC patients undergoing atezolizumab plus bevacizumab or other immunotherapies,^14^, ^15^, ^16^ its predictive capabilities in the context of HCC patients treated with TACE combined with lenvatinib remain unexplored. Notably, a recent study by Guan suggested that CRAFITY serves as a superior predictor for individuals with HCC undergoing TACE or hepatic arterial infusion chemotherapy in conjunction with immunotherapy.^15^ Those findings prompt the question of whether the CRAFITY score could potentially serve as a prognostic predictor for patients undergoing TACE combined with lenvatinib.

Consequently, the primary objective of this study is to assess the correlation between the CRAFITY score and the prognosis of patients with HCC treated with TACE and lenvatinib. Our findings aim to provide valuable insights for oncologists in clinical practice.

## 2 MATERIALS AND METHODS

### 2.1 Patient enrollment

The study encompassed a total of 172 individuals diagnosed with HCC, who underwent treatment with TACE in combination with lenvatinib at the Affiliated Cancer Hospital of Shandong First Medical University. These patients were included in the training cohort, with the enrollment period spanning from August 2019 to August 2022. In addition, 142 patients with unresectable HCC who were receiving TACE and lenvatinib at the Cancer Hospital, Chinese Academy of Medical Sciences, between January 2020 and January 2022 were enrolled as the validation cohort.

The baseline data of all patients were retrospectively obtained from their electronic medical records. Independent ethics committees from each institution authorized the study. This work was approved by the Ethics Committee of the Affiliated Cancer Hospital of Shandong First Medical University (SDTHEC 2023007012). Written informed consent was obtained from all patients before the operation, and all procedures were in accordance with the 1955 Declaration of Helsinki.

The inclusion criteria for this study comprised: (1) confirmation of HCC through histopathology or non‐invasive diagnostic standard in accordance with the American Association for the Study of Liver Diseases guidelines^17^; (2) patients diagnosed with unresectable HCC who underwent a cumulative treatment duration of minimum 8 weeks with TACE combined with lenvatinib; (3) individuals demonstrating sufficient hematologic and organ function, an Eastern Cooperative Oncology Group (ECOG) PS score of 0 or 1, and a Child‐Pugh score equal to or less than 7 points; (4) presence of at least one measurable lesion according to the modified Response Evaluation Criteria in Solid Tumors (mRECIST) criteria.

Exclusion criteria encompassed: (1) patients who had previously undergone other antitumor treatments; (2) absence of TACE or lenvatinib treatment; (3) lack of follow‐up imaging, alpha‐fetoprotein (AFP), and C‐reactive protein (CRP) data; (4) loss to follow‐up; (5) individuals suffering from any other systemic ailments, including ongoing infection and other cancers. Figure S1 illustrates our procedural flow.

### 2.2 Treatment procedures

TACE adhered to a previously documented procedure.^18^ The process initiated with the insertion of a 5 French catheter into the celiac trunk. Following this, a 2.7 French microcatheter was meticulously positioned into the blood supply artery, and the precision of its location was affirmed through angiography. An emulsion comprising epirubicin and iodized oil was then introduced. Furthermore, particulate embolic agents, such as gelatin sponge particles, were administered until an observable deceleration or standstill in blood flow was noted. This was indicated by the lack of tumor staining on successive imaging, signifying the conclusion of embolization. In the presence of a considerable arteriovenous fistula, embolization was carried out, utilizing large‐diameter gelatin particles or spring coils as supplementary embolic agents.

A comparative analysis between embolization and preoperative computed tomography (CT) images guaranteed the thorough embolization of all liver tumor lesions. In instances of incomplete embolization, extrahepatic blood supply vessels were identified and subsequently subjected to embolization. The determination of whether to initiate a repetition of TACE was contingent upon the outcomes of the imaging evaluation following two cycles of TACE. If subsequent imaging disclosed persistent active lesions in the liver and the condition of the patient permitted, consideration was given to repeating TACE. Nevertheless, termination of TACE was deemed necessary if operational conditions rendered it unfeasible or if TACE proved ineffectual. The patient was prescribed oral lenvatinib at a dosage of 12 mg/day (for those with a body weight >60 kg) or 8 mg/day (for those with a body weight <60 kg). To ensure tolerability, lenvatinib was initiated 3–5 days before the initial TACE. Dose adjustments, ranging from 8 mg, 4 mg, to 4 mg every other day, were permitted in response to lenvatinib‐related toxicities. Intravenous administration of PD‐1 inhibitors, including camrelizumab, sintilimab, and tislelizumab, at a dose of 200 mg occurred on the day after TACE, with subsequent PD‐1 administrations taking place every 3 weeks. In the event of the first‐line combination therapy proving ineffective, the adoption of second‐line systemic therapy or alternative palliative treatments was recommended. The selection of second‐line agents was determined based on medical discernment, toxicity status, and the availability of the drug. Crucial therapeutic decisions were arrived at through multidisciplinary debates involving medical oncologists, surgeons, and radiologists.

### 2.3 Endpoints and evaluations

The central endpoint of this investigation was OS, computed from the commencement of the initial oral administration of lenvatinib until death resulting from any cause or the conclusion of the latest follow‐up. Supplementary endpoints encompassed disease control rate (DCR), progression‐free survival (PFS), and objective response rates (ORR). Analyses of survival were concluded with the final follow‐up conducted on June 1, 2023.All participants were subjected to AFP, CRP, and contrast‐enhanced computed tomography (CE‐CT) assessments within a fortnight preceding antitumor therapy, followed by subsequent evaluations every 4–6 weeks. Dimensions of the cumulative longest diameters of target lesions and viable (enhancing) target lesions were gauged from baseline and follow‐up CE‐CT pictures, employing mRECIST.^19^, ^20^ The ultimate assessment outcomes were derived from evaluations of both target and non‐target lesions, executed independently by two radiologists.

### 2.4 CRAFITY score

The determination of the CRAFITY score relied on the values of AFP and CRP, both gathered prior to the initiation of antitumor therapy. In alignment with Scheiner's investigation,^14^ patients presenting with AFP levels ≥100 ng/mL at baseline or CRP levels ≥1 mg/dL were allotted 1 point each; otherwise, they received 0 points. The CRAFITY score was calculated as the sum of these two individual scores. To illustrate, a patient exhibiting AFP levels <100 ng/mL and CRP levels <1 mg/dL received a CRAFITY score of 0. A patient with either AFP ≥100 ng/mL or CRP ≥1 mg/dL was deemed a CRAFITY score of 1 point, while a patient displaying both AFP ≥100 ng/mL and CRP ≥1 mg/dL received a CRAFITY score of 2 points.

### 2.5 Statistical analysis

Statistical examination was carried out utilizing SPSS v26.0 (SPSS Inc., USA) and the R language. Baseline characteristics of participants were presented for categorical variables as frequencies and percentages. The chi‐squared test was employed to compare categorical variables. For continuous variables, the mean value accompanied by the standard deviation was utilized. Contrast between different groups was conducted using either an independent sample *t*‐test or the Mann–Whitney *U* test. Survival time was gauged using the Kaplan–Meier method, and variations in survival time were assessed through stratified log‐rank tests. COX univariate analysis was executed to identify potential independent variables (*p* < 0.1). Following the elimination of evident collinearity through linear regression, the selected independent variables were incorporated into COX multivariate analysis. All analyses adhered to a two‐sided approach, and statistical significance was established with a *p* < 0.05.

## 3 RESULTS

### 3.1 Baseline clinical data

This study enrolled 172 patients for the training cohort and 142 for the validation cohort. The majority of cases in the training cohort were male (144 individuals, constituting 83.7%), while in the validation cohort, 98 cases (69.0%) were male. Viral hepatitis B emerged as the primary cause of chronic liver disease, accounting for 89.5% in the training cohort and 88.7% in the validation cohort. The majority of patients in both cohorts exhibited Child‐Pugh A grade (84.3% in the training cohort, 96.5% in the validation cohort), cirrhosis (83.7% in the training cohort, 78.9% in the validation cohort), and BCLC C stage (77.9% in the training cohort, 65.5% in the validation cohort).

Furthermore, a substantial proportion of patients had HCC with tumors exceeding 7 cm (76.7% in the training cohort, 76.1% in the validation cohort), multiple intrahepatic lesions (70.9% in the training cohort, 56.3% in the validation cohort), and macrovascular invasion (57.0% in the training cohort, 50.0% in the validation cohort). Notably, extrahepatic metastasis was absent in a significant majority (66.9% in the training cohort, 70.4% in the validation cohort).

In comparison to the validation cohort, the training cohort exhibited a higher prevalence of patients with BCLC stage C (*p* = 0.014), multiple lesions (*p* = 0.007), and elevated AST levels (*p* = 0.047). Detailed baseline clinical characteristics of patients can be found in Table 1.

**TABLE 1:** Patient and baseline characteristics in training cohort and validation cohort.

Characteristics		Pooled cohort (*n* = 314)	Training cohort (*n* = 172)	Validation cohort (*n* = 142)	*p‐*value
Age (years)	<60	203 (64.6%)	105 (61.1%)	98 (69.0%)	0.142
	≥60	111 (35.4%)	67 (38.9%)	44 (31.0%)	
Gender	Female	72 (22.9%)	28 (16.3%)	44 (31.0%)	0.002
	Male	242 (77.1%)	144 (83.7%)	98 (69.0%)	
Child‐Pugh	A	282 (89.8%)	145 (84.3%)	137 (96.5%)	0.001
	B	32 (10.2%)	27 (15.7%)	5 (3.5%)	
Etiology	Hepatitis B	280 (89.2%)	154 (89.5%)	126 (88.7%)	0.820
	No‐ Hepatitis B	34 (10.8%)	18 (10.5%)	16 (11.3%)	
Cirrhosis	No	58 (18.5%)	28 (16.3%)	30 (21.1%)	0.271
	Yes	256 (81.5%)	144 (83.7%)	112 (78.9%)	
BCLC stage	B	87 (27.7%)	38 (22.1%)	49 (34.5%)	0.014
	C	227 (72.3%)	134 (77.9%)	93 (65.5%)	
Max size (cm)	<7.0	74 (23.6%)	40 (23.3%)	34 (23.9%)	0.886
	≥7.0	240 (76.4%)	132 (76.7%)	108 (76.1%)	
Number	Solitary	112 (35.7%)	50 (29.1%)	62 (43.7%)	0.007
	Multiple	202 (64.3%)	122 (70.9%)	80 (56.3%)	
Macrovascular invasion	No	145 (46.2%)	74 (43.0%)	71 (50.0%)	0.217
	Yes	169 (53.8%)	98 (57.0%)	71 (50.0%)	
Extrahepatic metastases	No	215 (68.5%)	115 (66.9%)	100 (70.4%)	0.499
	Yes	99 (31.5%)	57 (33.1%)	42 (29.6%)	
CRAFITY score	0	53 (16.9%)	28 (16.3%)	25 (17.6%)	0.862
	1	141 (44.9%)	76 (44.2%)	65 (45.8%)	
	2	120 (38.2%)	68 (39.5%)	52 (36.6%)	
Combined PD‐1	Yes	121 (38.5%)	64 (37.2%)	57 (40.1%)	0.595
	No	193 (61.5%)	108 (62.8%)	85 (59.9%)	
ALT (U/L)^a^	Median, (IQR)	44.01 (28.10–64.70)	38.00 (27.00–65.75)	42.00 (31.00–52.85)	0.142
AST (U/L)	Median, (IQR)	62.79 (47.10–80.40)	59.00 (48.00–82.50)	52.00 (39.40–75.05)	0.047
Albumin (g/dL)	Median, (IQR)	40.22 (36.70–43.60)	40.10 (35.70–42.70)	42.00 (36.80–44.90)	0.095
Total bilirubin (mg/dL)	Median, (IQR)	19.85 (12.60–23.82)	20.50 (12.80–25.50)	17.50 (13.15–23.80)	0.231

*Note*: Unless indicated, data are numbers of patients, and numbers in parentheses are percentages.

Abbreviations: ALT, alaninetransaminase; AST, aspartate aminotransferase.

^a^ Data are median (interquartile range).

### 3.2 The efficacy of the CRAFITY score in predicting PFS and OS

**FIGURE 1:**
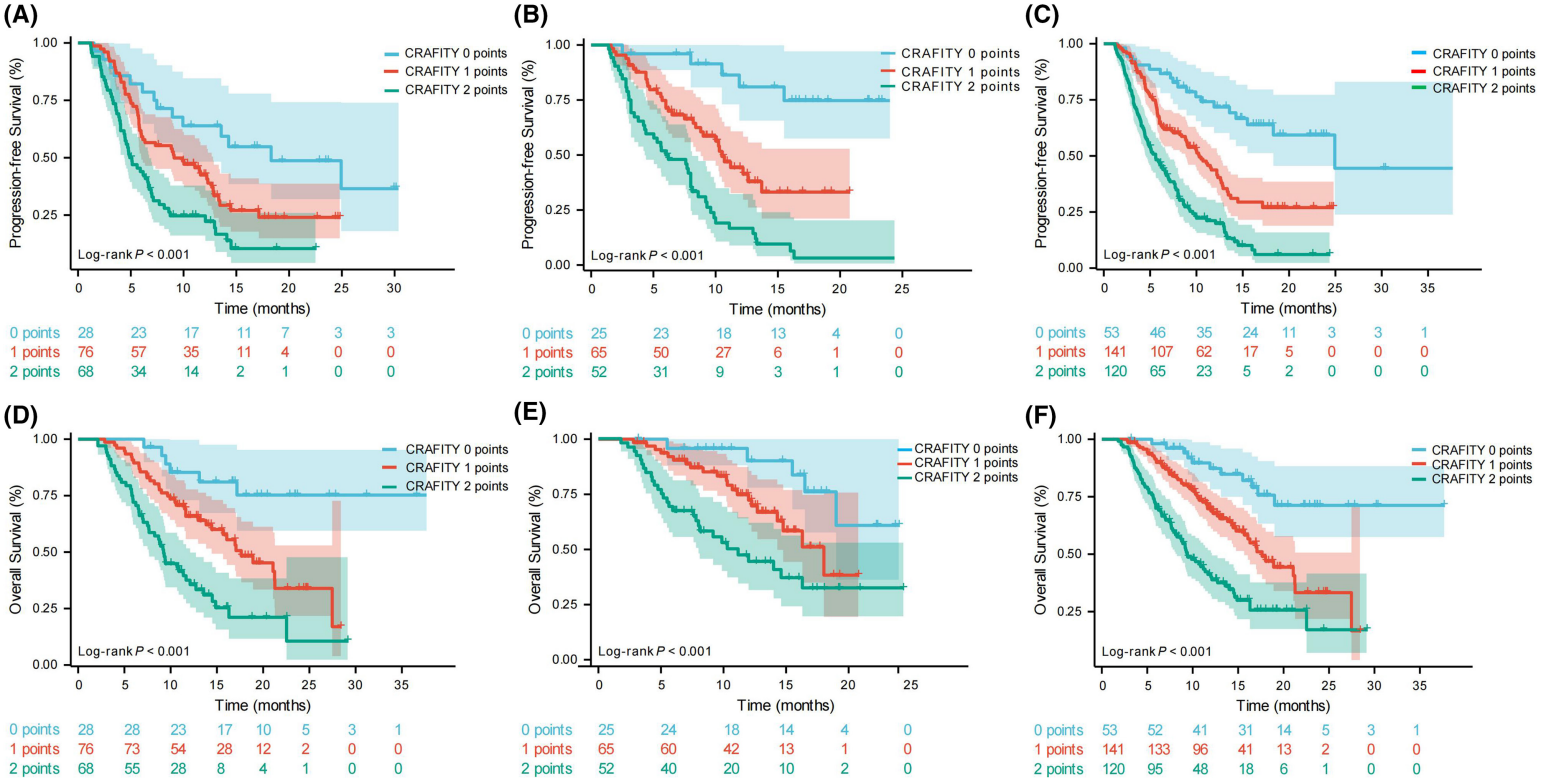
PFS and OS of HCC patients. PFS stratified of CRAFITY score in the training cohort (A), PFS stratified of CRAFITY score in the validation cohort (B), PFS stratified of CRAFITY score in the whole cohort (C), OS stratified of CRAFITY score in the training cohort (D), OS stratified of CRAFITY score in the validation cohort (E), OS stratified of CRAFITY score in the whole cohort (F). HCC, hepatocellular carcinoma; OS, overall survival; PFS, progression‐free survival.

At the conclusion of the follow‐up period, comprehensive records were available for 314 patients. In the training cohort, the PFS exhibited significant variation across the three groups according to the CRAFITY score (median PFS; 0 points group: 18.3 months [95% CI 8.1–28.5], 1 points group: 9.1 months [95% CI 4.9–13.3], 2 points group: 4.9 months [95% CI 3.6–6.2]; *p* < 0.001; see Figure 1A). Similarly, in the validation cohort, PFS demonstrated significant differences among the three groups according to the CRAFITY score (median PFS; 0 points group: not estimable [NE], 1 points group: 10.5 months [95% CI 9.2–11.8], 2 points group: 6.0 months [95% CI 8.5–11.5]; *p* < 0.001; refer to Figure 1B). When considering the entire cohort, the median PFS for CRAFITY scores of 0, 1, and 2 points was 24.9 months [95% CI 12.5–37.3], 10.3 months [95% CI 8.3–12.3], and 5.6 months [95% CI 4.4–6.8], respectively, showcasing a notable difference (*p* < 0.001; Figure 1C).

Within the training cohort, OS exhibited significant variability across the three groups stratified by the CRAFITY score (median OS; 0 points group: NE, 1 points group: 17.6 months [95% CI 12.9–22.4], 2 points group: 9.2 months [95% CI 8.4–10.0]; *p* < 0.001; refer to Figure 1D). Similarly, in the validation cohort, OS displayed noteworthy differences among the three groups as indicated by the CRAFITY score (median OS; 0 points group: NE, 1 points group: 18.0 months [95% CI 13.8–22.2], 2 points group: 11.2 months [95% CI 7.5–14.9]; *p* < 0.001; see Figure 1E). Considering the entire cohort, the median OS for CRAFITY scores of 0, 1, and 2 points was NE, 17.6 months [95% CI 15.1–20.2], and 9.4 months [95%CI 7.7–11.1], respectively, with a significant variation (p < 0.001; Figure 1F).

**FIGURE 2:**
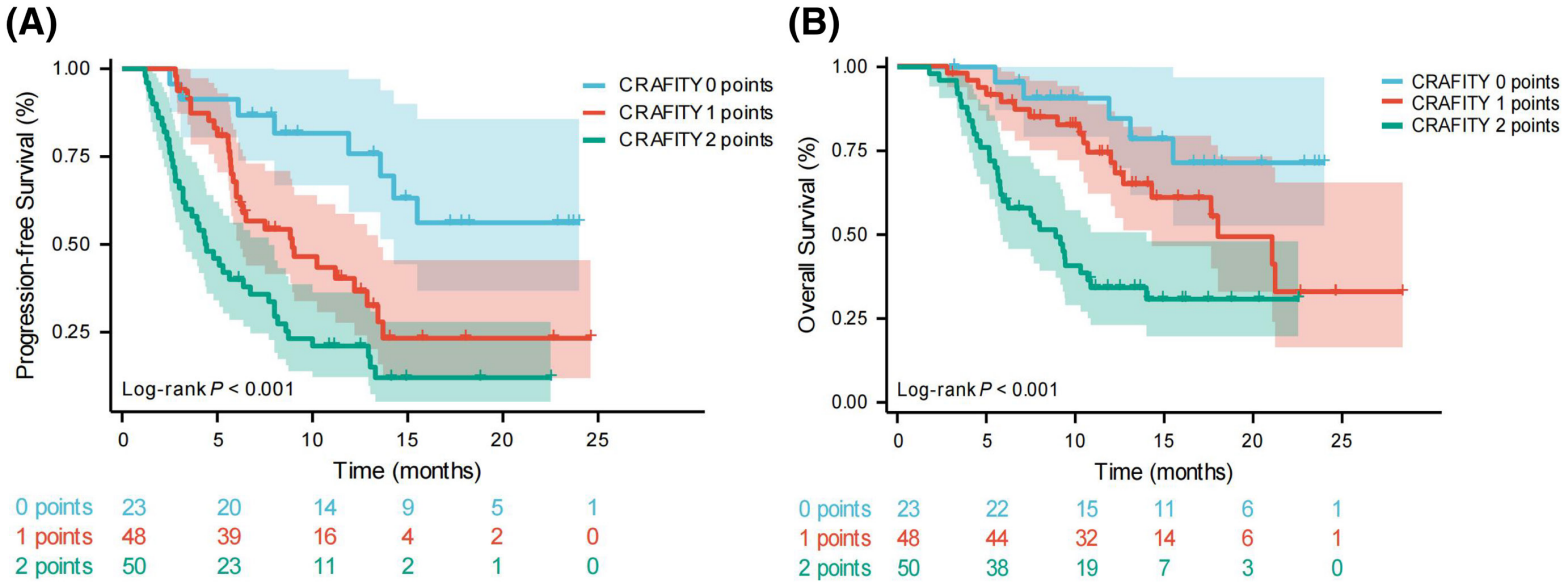
PFS and OS in 121 HCC patients treated with TACE, lenvatinib, and PD‐1. PFS stratified of CRAFITY score in 121 HCC patients (A), OS stratified of CRAFITY score in 121 HCC patients (B). HCC, hepatocellular carcinoma; OS, overall survival; PFS, progression‐free survival; TACE, transarterial chemoembolization.

Furthermore, we conducted an analysis specifically focusing on patients who underwent treatment with TACE, lenvatinib, and PD‐1 inhibitors (training cohort: 64 patients; validation cohort: 57 patients). The findings also demonstrated that the CRAFITY score effectively stratified the prognosis of those with HCC in the triple therapy group. Among the 121 patients, the median PFS for CRAFITY scores of 0, 1, and 2 was NE, 8.9 months (95% CI 4.8–13.1), and 4.4 months (95% CI 3.0–5.7), respectively, with a statistically significant difference observed among the groups (*p* < 0.001; see Figure 2A). Similarly, the median OS for the CRAFITY 0, 1, and 2 scores was NE, 18.0 months (95% CI 13.3–22.7), and 8.9 months (95% CI 6.8–11.0), respectively (*p* < 0.001; refer to Figure 2B) (for detailed PFS and OS information for the training and validation cohorts, please refer to Figure S2).

To mitigate the impact of immunotherapy, we conducted a more focused analysis on patients who received treatment with TACE in conjunction with lenvatinib (training cohort: 108 patients; validation cohort: 85 patients). In both PFS and OS, patients with a CRAFITY score of 0 exhibited significantly longer durations, followed by individuals with a CRAFITY score of 1, and subsequently, patients with a CRAFITY score of 2 points. This pattern held true across the training, validation, and entire cohorts (for detailed information, please refer to Figure S3).

### 3.3 Effectiveness of the CRAFITY score in forecasting ORR and DRC

Within the training cohort, CR, PR, stable disease (SD), and PD were observed in 20 (11.6%), 86 (50.0%), 42 (24.4%), and 24 (14.0%) patients, correspondingly. The ORR and DCR in the training cohort stood at 61.6% and 86.0%, respectively. When stratified by CRAFITY scores, the ORR for CRAFITY 0, 1, and 2 points were 78.6%, 68.4%, and 47.1%, respectively (*p* = 0.004). Correspondingly, the DCR for CRAFITY 0, 1, and 2 points were 96.4%, 85.5%, and 82.4%, respectively (*p* = 0.192).

In the validation cohort, CR, PR, SD, and PD were attained by 13 (9.2%), 66 (46.5%), 41 (28.9%), and 22 (15.5%) patients, respectively. The ORR and DCR in the validation cohort stood at 55.5% and 84.5%, respectively. When considering CRAFITY scores, the ORR for CRAFITY 0, 1, and 2 points were 76.0%, 56.9%, and 44.2%, correspondingly (*p* = 0.030). Likewise, the DCR for CRAFITY 0, 1, and 2 points were 100%, 92.3%, and 67.3%, respectively (*p* = 0.001). A comprehensive summary of the radiological assessment of treatment response in both the training and validation cohorts can be found in Table S1.

Moreover, the CRAFITY score demonstrated predictive capabilities for both ORR and DCR across the complete cohort. For CRAFITY 0 points versus CRAFITY 1 points versus CRAFITY 2 points, the ORR was 77.4% versus 63.1% versus 45.8% (*p =* 0.005), and the DCR resulted in 98.1% versus 88.7% versus 75.8% (*p =* 0.001) (a comprehensive overview of the clinical outcome for the entire cohort can be found in Table 2).

**TABLE 2:** Tumor response according to the CRAFITY score in the whole cohort.

Tumor response	mRECIST criteria				ORR	*p‐*value	DCR	*p‐*value
CRAFITY score	CR	PR	SD	PD	CR + PR		CR + PR + SD	
0 (*n* = 53)	8 (15.1%)	33 (62.2%)	11 (20.8%)	1 (1.9%)	41 (77.4%)	0.005	52 (98.1%)	0.001
1 (*n* = 141)	18 (12.8%)	71 (50.4%)	36 (25.5%)	16 (11.3%)	89 (63.1%)		125 (88.7%)	
2 (*n* = 120)	7 (5.8%)	48 (40.0%)	36 (30.0%)	29 (24.2%)	55 (45.8%)		91 (75.8%)	

Abbreviations: CR, complete response; DCR, disease control rate; mRECIST, modified Response Evaluation Criteria in Solid Tumors; PD, progressive disease; PR, partial response; SD, stable disease.

### 3.4 Univariable and multivariable analysis

The univariate and multivariate outcome of PFS and OS are shown in Table 3. N the univariate analysis, factors such as Child‐Pugh B, BCLC stage C, maximum tumor size ≥7 cm, presence of multiple tumors, macrovascular invasion, extrahepatic metastases, and CRAFITY scores of 1 and 2 points demonstrated significant associations with PFS. Multivariate analysis further revealed that Child‐Pugh B (HR 1.571; 95% CI, 1.043–2.367; *p* = 0.031), CRAFITY 1 points (HR 2.489; 95% CI 1.483–4.178; *p* = 0.001), CRAFITY 2 points (HR 4.080; 95% CI 2.400–6.938; *p* < 0.001) independently constituted risk factors for PFS.

**TABLE 3:** Cox regression analysis to predict PFS and OS in HCC patients treated with TACE and lenvatinib.

Variables	PFS				OS			
	Univariate analysis		Multivariate analysis		Univariate analysis		Multivariate analysis	
	HR (95% CI)	*p‐*value	HR (95% CI)	*p‐*value	HR (95% CI)	*p‐*value	HR (95% CI)	*p‐*value
Age (years)								
<60 vs. ≥60	0.535 (0.261–3.924)	0.251			0.513 (0.274–1.857)	0.365		
Gender								
Female vs. Male	0.516 (0.281–5.844)	0.372			0.427 (0.261–2.756)	0.471		
Child–Pugh								
A	Reference		Reference		Reference		Reference	
B	1.985 (1.322–2.980)	**0.001**	1.571 (1.043–2.367)	**0.031**	3.073 (2.008–4.702)	**<0.001**	2.561 (1.658–3.957)	**<0.001**
Etiology								
Other	Reference				Reference			
Hepatitis B	1.009 (0.636–1.601)	0.970			0.932 (0.554–1.567)	0.789		
Cirrhosis								
No vs. Yes	1.524 (0.712–2.823)	0.561			1.032 (0.635–2.693)	0.241		
BCLC stage								
B	Reference		Reference		Reference		Reference	
C	2.079 (1.481–2.918)	**<0.001**	1.382 (0.826–2.314)	0.218	2.293 (1.508–3.488)	**<0.001**	1.047 (0.557–1.969)	0.886
Max size (cm)								
<7.0 vs. ≥7.0	2.058 (1.429–2.964)	**<0.001**	1.448 (0.984–2.131)	0.061	2.696 (1.695–4.289)	**<0.001**	1.813 (1.114–2.951)	**0.017**
Number								
Solitary vs. Multiple	1.501 (1.115–2.021)	**0.007**	1.340 (0.992–1.811)	0.057	0.715 (0.502–1.018)	0.063	1.261 (0.882–1.803)	0.204
Macrovascular invasion								
No vs. Yes	1.706 (1.290–2.258)	**<0.001**	1.118 (0.743–1.681)	0.593	2.120 (1.509–2.979)	**<0.001**	1.664 (1.019–2.718)	**0.042**
Extrahepatic metastases								
No vs. Yes	1.399 (1.050–1.864)	**0.022**	1.209 (0.858–1.705)	0.278	1.512 (1.083–2.112)	**0.015**	1.530 (1.033–2.265)	**0.034**
CRAFITY score								
0	Reference		Reference		Reference		Reference	
1	2.660 (1.593–4.442)	**<0.001**	2.489 (1.483–4.178)	**0.001**	2.805 (1.468–5.360)	**0.002**	2.452 (1.275–4.717)	**0.007**
2	5.469 (3.283–9.112)	**<0.001**	4.080 (2.400–6.938)	**<0.001**	6.376 (3.362–12.092)	**<0.001**	4.037 (2.078–7.841)	**<0.001**

*Note*: Bold text indicates variable with *p* < 0.05.

The univariate analysis revealed significant associations between OS and various factors, including Child‐Pugh B, BCLC stage C, maximum tumor size ≥7 cm, macrovascular invasion, extrahepatic metastases, and CRAFITY scores of 1 and 2 points. In the multivariate analysis, independent risk factors for OS were identified as follows: Child‐Pugh B (HR 2.561; 95%CI 1.658–3.957; *p* < 0.001), tumor max size ≥7.0 cm (HR 1.813; 95% CI 1.114–2.951; *p* = 0.017), macrovascular invasion (HR 1.664; 95% CI 1.019–2.718; *p* = 0.042), extrahepatic metastases (HR 1.530; 95% CI, 1.033–2.265; *p* = 0.034), CRAFITY 1 points (HR 2.452; 95% CI 1.275–4.717; *p* = 0.007), and CRAFITY 2 points (HR 4.037; 95% CI 2.078–7.841; *p* < 0.001).

### 3.5 The effect of different CRAFITY scores on PFS and OS by subgroup

**FIGURE 3:**
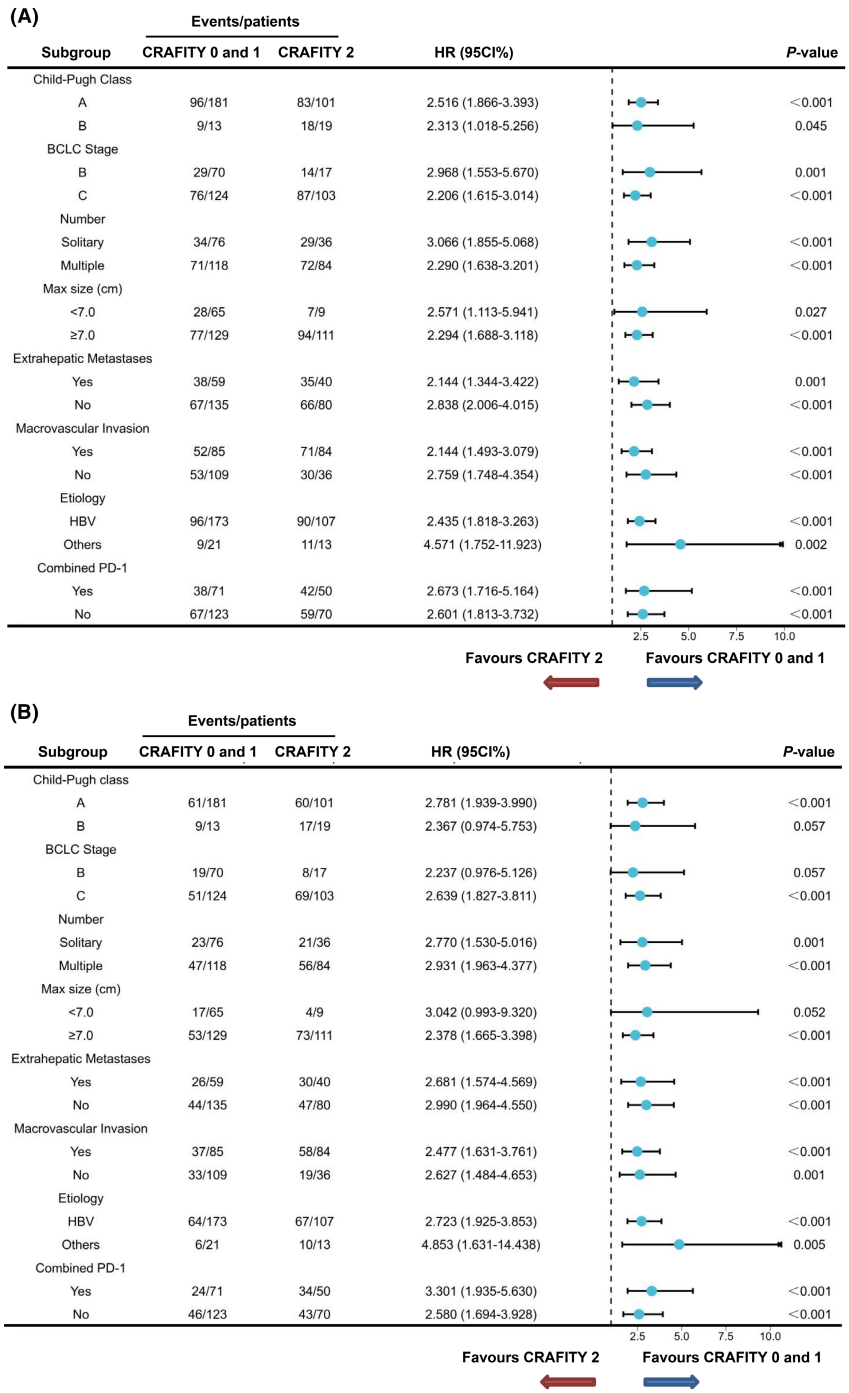
Median PFS (A), median OS (B), and hazard ratios for death comparing CRAFITY categories (0/1 vs. 2 points) in different subgroups in the entire cohort.

A detailed subgroup analysis was conducted, with each variable stratified to delve deeper into the impact of diverse CRAFITY scores on the outcomes of TACE plus lenvatinib. The results, depicted in Figure 3A, highlight that the CRAFITY 0 or 1 group consistently exhibited superior PFS across all subgroups. Furthermore, in Figure 3B, it is evident that the CRAFITY 0 or 1 group displayed improved OS compared to the CRAFITY 2 group within various subgroups. These subgroups encompassed patients with differing characteristics, including those with Child‐Pugh A or BCLC C stage, those with a single or multiple lesions, individuals with a maximum tumor size ≥7 cm, and those with or without extrahepatic metastases, macrovascular invasion, hepatitis B virus (HBV), and combined PD‐1 therapy.

## 4 DISCUSSION

Immunotherapy, specifically with immune checkpoint inhibitors, has demonstrated considerable efficacy against HCC. The US Food and Drug Administration has granted approval for the joint use of atezolizumab and bevacizumab in individuals grappling with unresectable or metastatic HCC undergoing their initial systemic treatment, as validated by the results from the phase III IMbrave150 study.^21^ Despite the strides made in enhancing the prognosis of those with advanced liver cancer through immunotherapy,^22^ it is imperative to recognize that only a specific portion of patients reaps the benefits of this therapeutic approach. Various biomarkers at molecular, transcriptional, genomic, and gut microbiome levels have been scrutinized to forecast the efficacy of immunotherapy.^23^, ^24^ Nevertheless, currently, a lack of validated biomarkers hampers their utility in guiding clinical decision‐making.

In a recent development, Scheiner et al. have introduced an innovative prognostic scoring system dubbed “CRAFITY,” anchored in serum CRP and AFP levels, specifically tailored to prognosticate outcomes in HCC patients treated with atezolizumab plus bevacizumab.^14^ The CRAFITY score adeptly sorts patients with HCC into three distinctive prognostic categories. A diminished CRAFITY score aligns with a more favorable tumor response and OS. Despite the noteworthy efficacy showcased by the atezolizumab/bevacizumab regimen, its financial burden renders it impractical for a significant proportion of HCC patients in China. Consequently, the widespread implementation of the “CRAFITY” model in China remains constrained. Lenvatinib has been endorsed as the primary treatment option for advanced HCC.^25^ In the phase III REFLECT trial, patients with advanced HCC who were administered lenvatinib experienced a substantial recovery in both PFS and ORR in comparison to those treated with sorafenib.^26^ However, the efficacy of lenvatinib remains suboptimal, evidenced by a median OS of 13.6 months. Integrating lenvatinib with a localized therapeutic approach targeting intrahepatic tumors, such as TACE, holds promise for enhancing outcomes in advanced HCC patients. A phase III randomized clinical trial (LAUNCH) proved that the addition of TACE to lenvatinib not only enhanced clinical outcomes but also emerged as a promising frontline treatment for individuals with late‐stage HCC.^5^

To our knowledge, there is a lack of a predictive model for anticipating the therapeutic effectiveness of the combined lenvatinib and TACE approach. Given the encouraging predictive outcomes observed with the CRAFITY score in the context of lenvatinib combined with TACE in this study, there is conjecture that CRAFITY could be employed to forecast the effectiveness of the combination therapy involving lenvatinib and TACE.

Based on the initial levels of AFP and CRP, a total of 174 patients diagnosed with HCC and undergoing treatment with lenvatinib and TACE were included in the training cohort. The participants were sorted into three groups based on their CRAFITY scores. The results revealed that the CRAFITY scores effectively predicted the patients' prognosis, with higher scores correlating with poorer OS (CRAFITY 0, 1, and 2 points: NE, 17.6 months, and 9.2 months; *p* < 0.001) and PFS (CRAFITY 0, 1, and 2 points: 18.3 months, 9.1 months, and 4.9 months; *p* < 0.001). These findings were subsequently validated in both an independent cohort and the entire cohort.

Moreover, in the training cohort, patients with HCC possessing a CRAFITY score of 0 exhibited a higher ORR compared to those with scores of 1 and 2. This trend was consistent in the validation and entire cohorts. Importantly, the CRAFITY score proved valuable not only for patients undergoing lenvatinib and TACE but also for those receiving TACE and a combination of lenvatinib plus PD‐1 inhibitors. In the context of triple therapy, the median PFS for CRAFITY scores of 0, 1, and 2 points was NE, 8.9 months, and 4.4 months, respectively (*p* < 0.001). Similarly, the median OS for CRAFITY scores of 0, 1, and 2 points was NE, 18.0 months, and 8.9 months, respectively (*p* < 0.001). Subgroup analysis further affirmed the accuracy of the CRAFITY score in stratifying the prognosis of HCC patients undergoing TACE, lenvatinib, and PD‐1 inhibitors. These results align partially with those reported in previous studies. The CRAFITY score has demonstrated a remarkable ability to predict the outcomes of HCC patients undergoing TACE and lenvatinib treatment. This proficiency is likely linked to its close association with the tumor immune microenvironment and inherent biological characteristics. CRP, an acute‐phase protein synthesized by the liver, serves as a nonspecific indicator of inflammatory activity. It is believed to mirror systemic and local inflammation, tumor invasiveness, and the systemic dissemination of cancer cells.^27^, ^28^ Tumor growth induces tissue inflammation, including IL‐6, which elevates CRP levels.^29^ CRP may engage with immunosuppressive cells, such as CD68+ tumor‐associated macrophages and CD15+ tumor‐associated neutrophils (TANs), contributing to a suppressed tumor immune microenvironment.^30^ A retrospective analysis by Hayashi et al. involving 53 unresectable HCC patients treated with lenvatinib indicated that the OS of the high CRP group was significantly lower than that of the low CRP group.^31^ Additionally, CRP, when combined with albumin levels, erythrocyte sedimentation rate, neutrophil‐to‐lymphocyte ratios, and other factors, has been widely employed to predict tumor prognosis.^28^, ^32^

AFP a mammalian fetal glycoprotein associated with tumors, stands out as one of the most commonly used serological markers for HCC. It holds significant research value in understanding the occurrence, development, diagnosis, and treatment of HCC.^33^ Elevated AFP is linked to inhibiting the programmed cell death of tumor cells, promoting their proliferation and metastasis, inhibiting the functions of lymphocytes, macrophages, and dendritic cells, inducing malignant transformation, and facilitating the onset of HCC. Numerous studies have corroborated that elevated AFP is associated with treatment resistance, poor prognosis, and serves as a critical negative regulatory factor for HCC.^34^, ^35^

Nevertheless, this study has certain limitations that warrant acknowledgment. First, being a retrospective study, inherent information and selection biases may be present. Second, the observation period in the current investigation was relatively short, underscoring the need for further prospective studies with extended follow‐up durations. Third, the study was conducted at only two centers and with a relatively small sample size. In summary, to substantiate the predictive value of the CRAFITY score in patients with HCC undergoing TACE and lenvatinib, a multicenter, randomized, controlled clinical trial with a larger cohort seems imperative. In summary, our study illustrated that the CRAFITY score, utilizing AFP and CRP levels, is capable of forecasting the efficacy of TACE combined with lenvatinib in individuals with unresectable HCC. This score, easily obtainable in clinical practice, has the potential to aid oncologists in making informed clinical decisions. Nevertheless, further validation is crucial, particularly in the context of large sample size multicenter cohorts.

## AUTHOR CONTRIBUTIONS

**Lin Zhang:** Validation (equal); writing – original draft (equal). **Hongcai Yang:** Investigation (equal); writing – original draft (equal). **Shangkun Ning:** Data curation (equal); software (equal); visualization (equal). **Zhijuan Wu:** Validation (equal); visualization (equal). **Dianzhe Wang:** Validation (equal). **Hexin Liang:** Validation (equal). **Chunni Wang:** Resources (equal); supervision (equal). **Xu Chang:** Conceptualization (lead); resources (equal); supervision (equal); writing – review and editing (lead).

## FUNDING INFORMATION

This research was supported by Natural Science Foundation of Shandong Province (Grant number ZR2020QH177), The Youth Fund from Natural Science Foundation of Shandong Province (Grant number ZR2020QH244) and Shandong Traditional Chinese Medicine Science and Technology Project (Grant number Q‐2022110).

## CONFLICT OF INTEREST STATEMENT

Lin Zhang, Hongcai Yang, Shangkun Ning, Zhijuan Wu, Dianzhe Wang, Hexin Liang, Chunni Wang, and Xu Chang declare that they have no conflict of interest.

## ETHICS STATEMENT

All procedures followed were in accordance with the Helsinki Declaration of 1975, as revised in 2008.Informed consent was obtained from all patients for being included in the study.

## CONSENT

Written informed consent for publication of this study was obtained from the patient. A copy of the written consent form is available for review by the Editor of this journal.

## Supporting information


Data S1:

## Data Availability

The dataset used for this study is available from the corresponding author upon reasonable request.
