# Supplementary material for: CRAFITY score benefits hepatocellular carcinoma patients treated with transarterial chemoembolization and lenvatinib
Source: Cancer Med. 2024 Jun 24;13(12):e7410. doi: 10.1002/cam4.7410 (PMC11194610; doi:10.1002/cam4.7410)
Supplement: Supplementary file 1 — Data S1: [file CAM4-13-e7410-s001.docx]

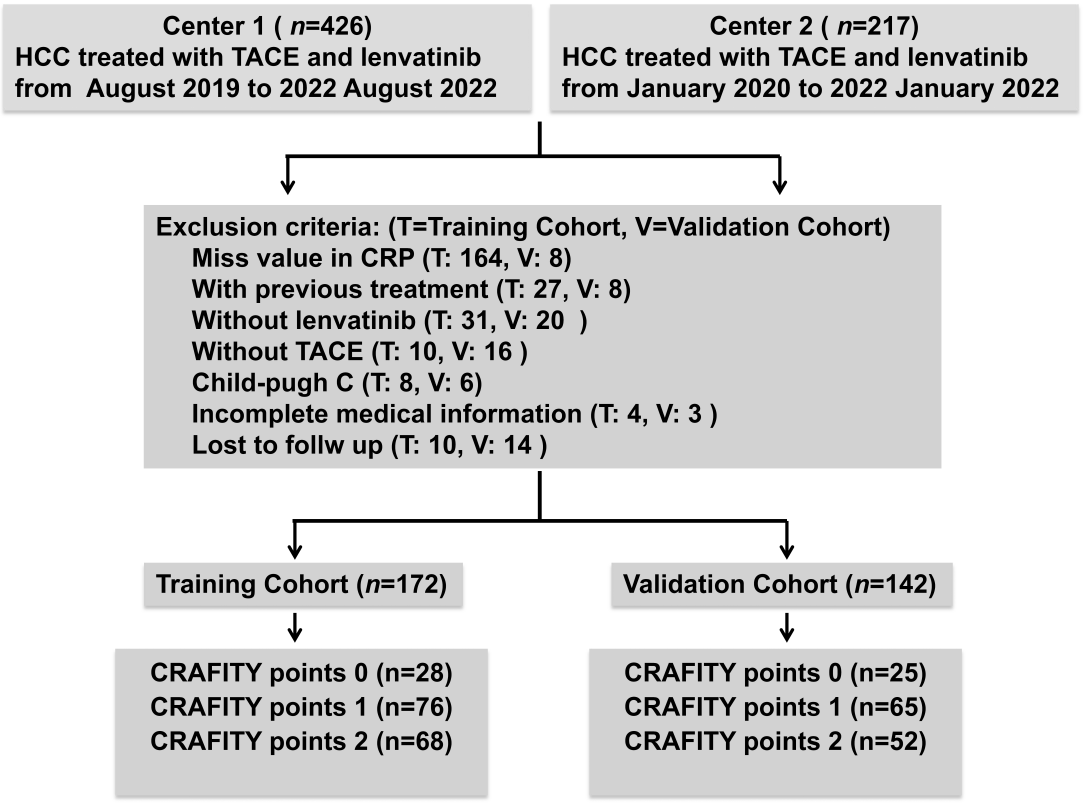


**Supplementary Figure 1.**Flowchart of the patient cohort.


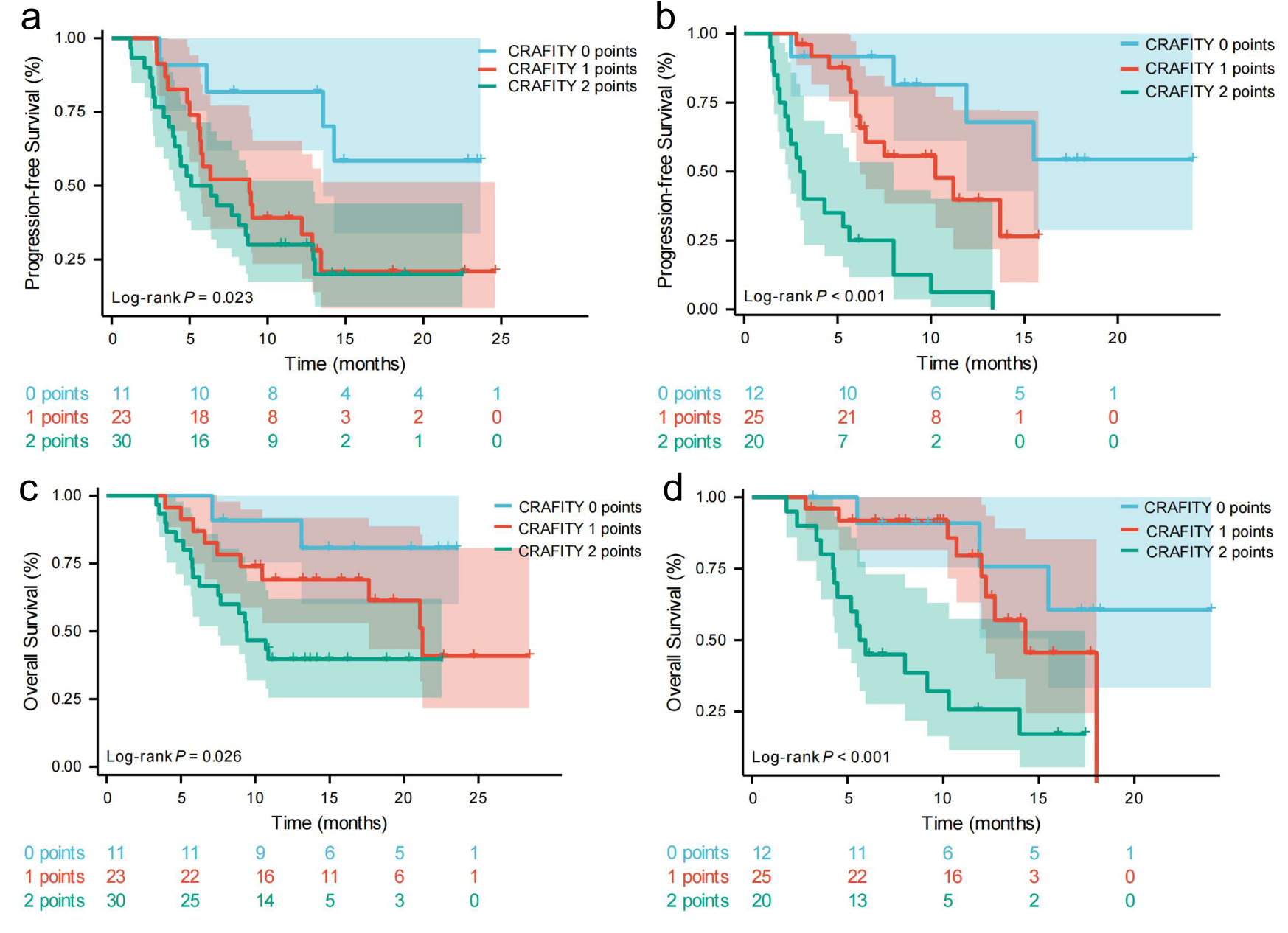


**Supplementary Figure2**. PFS and OS in 121 HCC patients treated with TACE, lenvatinib, and PD-1. (**a**) PFS stratified CRAFITY score in the training cohort. The median PFS of the CRAFITY 0, 1, and 2 scores was NE, 8.8 months (95%CI 4.0–13.7), and 5.1 months (95%CI 2.0–8.2), respectively (p< 0.001).(**b**) PFS stratifed of CRAFITY score in the validation cohort. The median PFS of the CRAFITY 0, 1, and 2 scores wasNE, 10.2 months (95%CI 4.9–15.5), and 3.0 months (95%CI 2.4–3.6), respectively (p< 0.001). (**c**) OS stratified of CRAFITY score in the training cohort. The median OS of the CRAFITY 0, 1, and 2 scores wasNE, 21.2 months (95%CI 16.4–26.1), and 9.4 months (95%CI 11.2–30.9), respectively (p< 0.001). (**d**) OS stratifed of CRAFITY score in the validation cohort. The median OS of the CRAFITY 0, 1, and 2 scores wasNE, 14.3 months (95%CI 11.6–17.0), and 5.6 months (95%CI 4.7–6.5), respectively (p< 0.001).


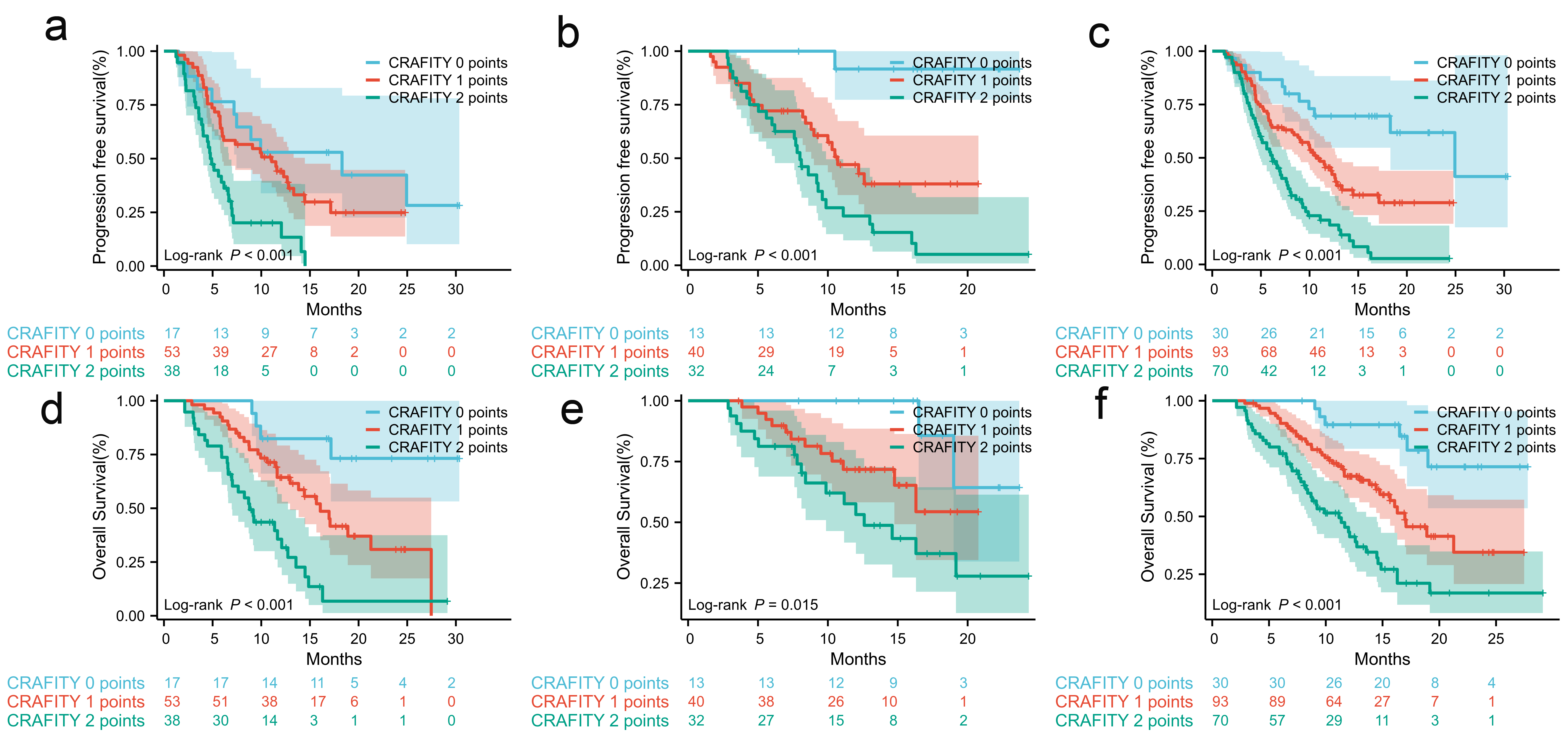


**Supplementary Figure3**. PFS and OS of HCC patients treated with TACE plus lenvatinib and without PD-1. (**a**) PFS stratified CRAFITY score in the training cohort. The median PFS in the patients with CRAFITY scores of 0, 1, and 2 points was 18.3 months (95%CI 3.1-33.4), 11.0 months (95%CI 6.4-15.7), and 4.7 months (95%CI 3.9-5.6), respectively(*p*<0.001). (**b**) PFS stratified of CRAFITY score in the validation cohort. The median PFS in the patients with CRAFITY scores of 0, 1, and 2 points was NE, 10.7 months (95%CI 8.1-13.2), and 8.0 months (95%CI 6.8-9.1), respectively(*p*<0.001). (**c**) PFS stratified of CRAFITY score in the whole cohort. The median PFS in the patients with CRAFITY scores of 0, 1, and 2 points was 24.9 (95%CI 13.4-36.4), 10.7 months (95%CI 8.7-12.6), and 6.1 months (95%CI 4.6-7.7), respectively(*p*<0.001). The PFS was significantly longer in the patients with a CRAFITY score of 0, followed by those with a CRAFITY score of 1 and those with a CRAFITY score of 2 points (*p*<0.001). (**d**) OS stratified of CRAFITY score in the training cohort. The median OS was NE in patients with CRAFITY scores of 0, while it was 16.0 months (95%CI 13.0-19.0) in patients with CRAFITY score of 1 and 8.8 months (95%CI 6.9-10.6) in patients with CRAFITY score of 2 (*p*<0.001). (**e**) OS stratified of CRAFITY score in the training cohort. The median OS was NE in patients with CRAFITY scores of 0 and 1, while it was 12.0 months (95%CI 7.9-17.2) in patients with CRAFITY scores of 2 (*p*=0.015). (**f**) OS stratified of CRAFITY score in the whole cohort. The median OS was NE in patients with CRAFITY scores of 0, while it was 16.9 months (95%CI 14.1-19.7) in patients with CRAFITY score of 1 and 11.2 months (95%CI 8.2-14.1) in patients with CRAFITY score of 2(*p*<0.001). PFS, progression-free survival; OS, overall survival; HCC. hepatocellular carcinoma.NE; is not estimable.

| **Supplementary Table 1.**Tumor response according to the CRAFITY score in the training and validation cohort. | | | | |
| --- | --- | --- | --- | --- |
| **Variable** | **CRAFITY 0** | **CRAFITY 1** | **CRAFITY 2** | ***P*-value** |
| **Training Cohort (n=172)** | n=28 | n=76 | n=68 |  |
| CR | 6 (21.4%) | 11 (14.5%) | 3(4.4%) |  |
| PR | 16 (57.1%) | 41 (53.9%) | 29(42.6%) |  |
| SD | 5 (17.9%) | 13 (17.1%) | 24(35.3%) |  |
| PD | 1 (3.6%) | 11 (14.5%) | 12(17.6%) |  |
| ORR | 22 (78.6%) | 52 (68.4%) | 32(47.1%) | 0.004 |
| DCR | 27 (96.4%) | 65 (85.5%) | 56 (82.4%) | 0.192 |
| **Validation Cohort (n=142 )** | n=25 | n=65 | n=52 |  |
| CR | 2 (8.0%) | 7 (10.8%) | 4 (7.7%) |  |
| PR | 17 (68.0%) | 30 (46.1%) | 19 (36.5%) |  |
| SD | 6 (24.0%) | 23 (35.4%) | 12 (23.1%) |  |
| PD | 0 | 5 (7.7%) | 17 (32.7%) |  |
| ORR | 19 (76%) | 37 (56.9%) | 23 (44.2%) | 0.030 |
| DCR | 25 (100%) | 60 (92.3%) | 35 (67.3%) | 0.001 |
| Note: CR, Complete response; PR, Partial response; SD, Stable disease; PD, Progressive disease. | | | | |

.
